# Complement C5a receptor 1 antagonist attenuates alveolar hypoplasia induced by pulmonary hypoperfusion and its underlying mechanisms

**DOI:** 10.3389/fimmu.2026.1802250

**Published:** 2026-05-11

**Authors:** Chenxi Liu, Sixie Zheng, Ye Wang, Ziwen Wang, Debao Li, Hao Li, Yiting Xue, Zheng Wang, Siqi She, Lincai Ye, Peisen Ruan, Lijun Fu, Qi Sun

**Affiliations:** 1 Department of Rheumatology and Immunology, Shanghai Children’s Medical Center, Shanghai Jiao Tong University School of Medicine, Shanghai, China; 2 Department of Thoracic and Cardiovascular Surgery, Shanghai Children’s Medical Center, Shanghai Jiao Tong University School of Medicine, Shanghai, China; 3 Institute of Pediatric Translational Medicine, Shanghai Children’s Medical Center, Shanghai Jiao Tong University School of Medicine, Shanghai, China; 4 Department of Thoracic and Cardiovascular Surgery, Women and Children’s Health Care Hospital of Linyi, Linyi, Shandong, China; 5 Department of Pediatric Surgery, Children’s Hospital of Fudan University, National Children’s Medical Center, Shanghai, China; 6 Shanghai Institute for Pediatric Congenital Heart Disease, Shanghai Children’s Medical Center, Shanghai Jiao Tong University School of Medicine, Shanghai, China; 7 Department of Pediatric Critical Care Medicine, The Affiliated Women and Children’s Hospital of Ningbo University, Ningbo, Zhejiang, China; 8 Department of Cardiology, Shanghai Children’s Medical Center, Shanghai Jiao Tong University School of Medicine, Shanghai, China

**Keywords:** alveolar hypoplasia, C5aR1, congenital heart disease, IL-1β, pulmonary hypoperfusion

## Abstract

**Background:**

To elucidate the role of complement component C5a in pulmonary hypoperfusion (PHypo)-induced alveolar hypoplasia, given its centrality in sterile inflammation and the recent implication of immune activation in a neonatal rat PHypo model.

**Methods:**

PHypo was induced in postnatal day 1 (P1) rats via pulmonary artery banding (PAB). Bulk RNA sequencing of lung tissues was performed at P7 and P14, and Single-cell RNA sequencing was conducted at P7. Alveolar development was assessed histologically (H&E) at P14. PHypo rats were treated with a C5a receptor 1(C5aR1) antagonist (C5aR1 ant) at P1. Key molecular changes (IL-1β, NF-κB, SEMA3a) and alveolar cell number were evaluated. Serum C5a levels were measured in children with PHypo versus controls.

**Results:**

Bulk RNA-sequencing of P7 lungs identified the complement cascade in the top 20 pathway enrichment, with C5aR1 as the most significantly upregulated gene. Single-cell RNA sequencing revealed that C5aR1 and IL-11β are predominantly expressed in monocytes/macrophages, and immunofluorescence further demonstrated cytoplasmic localization of C5aR1. PHypo induced notable alveolar hypoplasia at P14. RNA-sequencing of P14 lungs showed downregulation of genes associated with alveolar formation and upregulation of those related to immune responses in PHypo lungs. Treatment with C5aR1 ant rescued PHypo-induced alveolar hypoplasia. Mechanistically, C5a-induced IL-1β activated NF-κB, inhibiting SEMA3a, crucial for alveolar budding. C5aR1 ant downregulated IL-1β/NF-κB axis and upregulated SEMA3a; direct inhibition of IL-1β replicated these effects and improved alveologenesis.

**Conclusions:**

This study defines a C5a–IL-1β–NF-κB–SEMA3a axis driving PHypo-induced alveolar hypoplasia. C5aR1 antagonist and IL-1β inhibitors demonstrate therapeutic efficacy, may offer novel strategies to improve long-term outcomes in affected children.

## 1 Introduction

Congenital heart disease (CHD) represents the most prevalent birth defect, with children exhibiting pulmonary hypoperfusion (PHypo) demonstrating significantly reduced exercise tolerance in adulthood, severely compromising their quality of life (1–5). Although advances in surgical techniques have markedly improved survival rates in pediatric CHDs, long-term exercise intolerance persists in adult survivors (1–5). Clinical studies indicate that diminished lung volume and impaired alveolar development in PHypo-CHD children critically contribute to this exercise deficit, though the underlying molecular mechanisms remain undefined (6). A key research barrier has been the absence of animal models replicating PHypo during the critical human alveolar developmental window (postnatal 0–7 years), hindering mechanistic investigation into PHypo-induced alveolar impairment (1, 7–9). Developing neonatal PHypo models and elucidating these mechanisms is essential to address this knowledge gap and advance therapeutic strategies for long-term patient outcomes.

Genetically edited PHypo models often exhibit severe concurrent malformations, resulting in embryonic lethality, while pharmacologically induced neonatal PHypo models or organoids models remain unavailable (7–9). Consequently, surgical induction currently represents the only feasible approach (7–9). Clinically, right ventricular outflow tract obstruction (RVOTO) constitutes the primary CHD etiology driving PHypo (7–9). Pulmonary artery banding (PAB)—surgically creating RVOTO—thus serves as a physiologically relevant PHypo model (7, 8). Using neonatal rat PHypo models and human RVOTO lung specimens, our prior work implicated activated immune-inflammatory responses in PHypo-driven pulmonary hypoplasia, though their precise role in restricting alveologenesis remains unclear (9).

Alveolar formation involves budding from the bronchial tree through axon guidance-like mechanisms (10–15). Recent studies highlight complement C5a as a core mediator of sterile inflammation (16–19). Monocytes/macrophages secrete C5a in an autocrine manner, binding C5a receptor 1(C5aR1) to shift energy metabolism from oxidative phosphorylation to glycolysis. Resulting glycolytic intermediates transcriptionally upregulate IL-1β (16). In neural systems, IL-1β binding to IL-1R1 triggers phosphorylation of NF-κB p65 at Ser536 (phospho-S536-p65), suppressing the axon guidance molecule SEMA3a (20, 21). Whether IL-1β similarly modulates alveolar development is unknown. This study therefore aims to: (1) establish a neonatal rat PHypo model, and (2) employ transcriptomics and pharmacologically interventional approaches to define the role and mechanisms of C5a in PHypo-induced alveolar hypoplasia.

## 2 Materials and methods

### 2.1 Data availability statement

The data generated in this study are available from the corresponding author upon reasonable request. The bulk RNA-seq data have been deposited in the GEO database (https://www.ncbi.nlm.nih.gov/geo) under accession No. GSE201522 and No. GSE206972, respectively.

### 2.2 Study design

**Figure 1:**
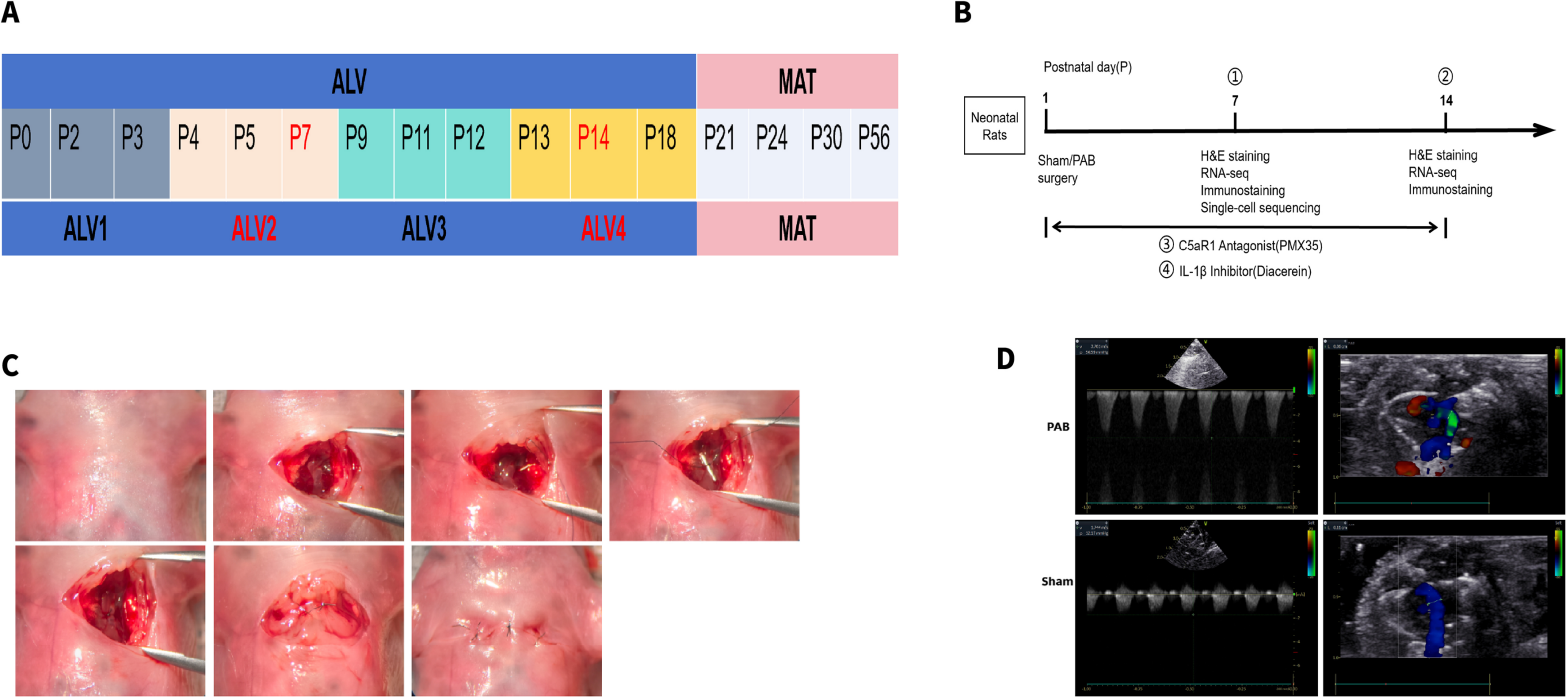
PAB modeling and experimental design. **(A)** The lung development cycle of rodents. **(B)** Experimental Procedure. **(C)** The surgical procedure for constriction of PAB in newborn rat. **(D)** Pulmonary artery blood flow and its Doppler spectrum.

Given the defined four-stage progression of rodent alveolar development—alveolar stage 1 (ALV1, postnatal day (P) 0-P3), ALV2 (P4-P7), ALV3 (P9-P12), and ALV4 (P13-P18) (**Figure 1A**)—we implemented a phased experimental approach (**Figure 1B**): P7 was selected to capture early molecular events preceding alveolar simplification, while P14 was used to confirm the persistence of structural changes. Therapeutic intervention with the C5a receptor 1(C5aR1) antagonist PMX53 (0.5mg/kg, intraperitoneal injection) effectively rescued PHypo-induced alveolar hypoplasia; and mechanistic validation was achieved through modulation of the downstream effector IL-1β.The primary endpoints for assessing alveologenesis in this study were: (1) mean linear intercept (MLI) as a measure of alveolar airspace size; (2) immunofluorescence quantification of AT1 (Rage) and AT2 (Sftpc) cells; and (3) expression of SEMA3a, a key regulator of alveolar budding. These endpoints were consistently applied across intervention groups.

### 2.3 PAB surgery

Pregnant Sprague-Dawley rats (BiKai Laboratory Animal Co., Ltd., Shanghai, China) were individually housed under controlled conditions (22 ± 1 °C, 12:12 light-dark cycle) with *ad libitum* access to food and water. Pups underwent PAB or sham surgery as previously described (**Figure 1C**) (7). Neonatal rats were anesthetized via hypothermic induction (3-min exposure on crushed ice) and maintained in a supine position on a cooling pad. A transverse skin incision was made at the third intercostal space followed by blunt dissection to enter the thoracic cavity. After pericardial retraction, the main pulmonary artery (PA) was isolated. A 12–0 nylon suture was passed beneath the artery and ligated against a 30-gauge needle. Subsequent needle removal created a calibrated stenosis matching the needle diameter. The thorax was closed in layers. Postoperatively, pups recovered on a 37 °C thermostatic warming platform until resumption of spontaneous movement (typically 15–20 min), followed by maternal reunion. Sham-operated controls received identical procedures excluding PA ligation.

### 2.4 Ultrasound

Transthoracic echocardiography was performed by a single blinded echocardiologist to quantify pressure gradients and blood flow across the PA. At postnatal day 7 (P7), rats underwent anesthesia induction (5% isoflurane in oxygen for 3–5 min) followed by maintenance (2.5% isoflurane via nasal cone) on a thermostatically controlled warming platform (37 °C). Cardiac parameters were acquired using a Vevo 2100 system (VisualSonics, Toronto) with a 25-MHz MS400 transducer. Pulsed-wave Doppler measurements were obtained in the parasternal long-axis view to assess: PA blood flow velocity-time integrals (VTI) and peak pressure gradient (PPG) across the PA.

### 2.5 RNA isolation and quantitative PCR

Total RNA was extracted from lung tissue using the PureLink™ RNA Micro Scale Kit (Thermo Fisher Scientific, Cat# 12183016), with RNA integrity verified (RIN > 8.0). Reverse transcription was subsequently performed using the PrimeScript™ RT Reagent Kit (Takara Bio). Quantitative PCR analysis employed SYBR™ Green PowerUp™ Master Mix (Applied Biosystems) on a 7900HT Fast Real-Time PCR System under optimized thermal cycling conditions: initial denaturation at 95 °C for 10 min followed by 40 cycles of 95 °C for 15 s and 60 °C for 60 s. Gene-specific primers (Generay Biotech; sequences provided in **Supplementary Table S1**) validated for 90-110% amplification efficiency were used, and relative gene expression was calculated via the 2^(-ΔΔCT) method normalized to GAPDH reference genes.

### 2.6 Bulk RNA-seq analysis

Sequencing libraries were prepared with the NEBNext^®^ Ultra™ II RNA Library Prep Kit (NEB, E7760) following manufacturer’s protocols. Libraries underwent 150-bp paired-end sequencing on an Illumina NovaSeq 6000 platform (Novogene Co., Ltd.). Raw FASTQ files were processed through in-house PERL scripts to produce high-quality clean data. Differential expression analysis was performed using DESeq2 (v1.38.3) with adjusted p-values (Benjamini-Hochberg FDR) < 0.05 defining significance. Functional enrichment analyses (Gene Ontology, Kyoto Encyclopedia of Genes and Genomes) were conducted via clusterProfiler (v4.8.1) with significant terms (FDR < 0.05) visualized in R. All analyses were executed on the OEbiotech Cloud Platform (https://cloud.oebiotech.com).

### 2.7 Single-cell RNA sequencing data processing and analysis

Single-cell transcriptomic data were analyzed using the Seurat R package (v5.3.0) (22). To ensure data quality, a multi-step quality control pipeline was implemented. Specifically, cells with fewer than 200 or more than 8,000 detected genes, as well as those with total unique molecular identifier (UMI) counts below 500 or above 25,000, were excluded. In addition, cells with a mitochondrial gene fraction greater than 20%, erythrocyte-specific gene expression exceeding 1%, or low transcriptomic complexity (log10GenesPerUMI < 0.7) were removed. Potential doublets were subsequently identified and excluded using the scDblFinder R package (v1.20.2) (23). For downstream analyses, data from different samples were integrated and batch effects were corrected using the Harmony algorithm (24). Principal component analysis (PCA) was first performed for linear dimensionality reduction, followed by uniform manifold approximation and projection (UMAP) for non-linear visualization and unsupervised clustering. Cluster-specific marker genes were identified using the FindAllMarkers function, with a minimum detection rate of 0.25 and a log fold-change threshold greater than 0.25. Cell types were then systematically annotated using the SingleR package with reference to the CellMarker 2.0 database. Gene set scores were calculated using the AddModuleScore function in Seurat.

### 2.8 H&E staining and alveolar morphometry

Hematoxylin and eosin (H&E) staining was performed to evaluate alveolarization using an H&E staining kit (Solarbio, Shanghai, China) according to routine protocols and was imaged under an optical microscope. Alveolar development was quantified using the mean linear intercept (MLI) method, a validated index of mean airspace diameter (25, 26). Following systematic random sampling, lung sections (5 μm, H&E-stained) were imaged at 200× magnification. For each terminal bronchiole-acinar unit, a crosshair centered on the bronchiole lumen was superimposed. The total length of the horizontal and vertical lines (typically 500 μm each) and the number of alveolar septal intersections were recorded. MLI (μm) was calculated as: MLI = Total line length (μm)/Number of septal intersections.

### 2.9 Immunofluorescence

The lungs were sectioned and subjected to immunofluorescence staining. The slides were incubated with primary antibodies (**Supplementary Table S1**) overnight at 4 °C. The slides were then incubated with secondary antibodies and 4’,6-diamidino-2-phenylindole (DAPI) for 30 min. ImageJ software (NIH, Bethesda, MD, USA) was used for quantification.

### 2.10 Serum cytokines determination

Blood samples from pediatric patients were centrifuged at 3,000 × g for 20 minutes, and the resulting serum was aliquoted after 2-fold dilution and stored at -80 °C. C5a concentrations were quantified using a commercial ELISA kit (PC094, Beyotime Biotechnology) according to the manufacturer’s protocol. Similarly, IL-1β levels were measured using a specific ELISA kit (ab214025, Abcam) following the manufacturer’s instructions.

### 2.11 Statistical analysis

Continuous variables are expressed as mean ± SD. Normality was assessed using Shapiro-Wilk tests (α=0.05). Parametric data underwent unpaired two-tailed t-tests (two groups) or one-way ANOVA with Šidák *post hoc* correction (≥3 groups). Non-parametric equivalents (Mann-Whitney U or Kruskal-Wallis/Dunn’s tests) were applied where appropriate. All analyses used SAS 9.4 (PROC GLIMMIX, SAS Institute), with significance defined as p<0.05 unless otherwise specified. We define the meanings of the difference indicators as follows: *: P ≤ 0.05; **: P ≤ 0.01; ***: P ≤ 0.001; ns: P > 0.05.

## 3 Results

### 3.1 PHypo activates C5aR1 signaling at ALV2

As shown in **Figures 1B,C**, neonatal rats underwent PAB surgery at P1. Echocardiography at performed at P7 revealed turbulent flow at the banding site with significantly increased transpulmonary velocity-time integral and pressure gradient in the PAB group compared with the sham group (**Figure 1D**).

**Figure 2:**
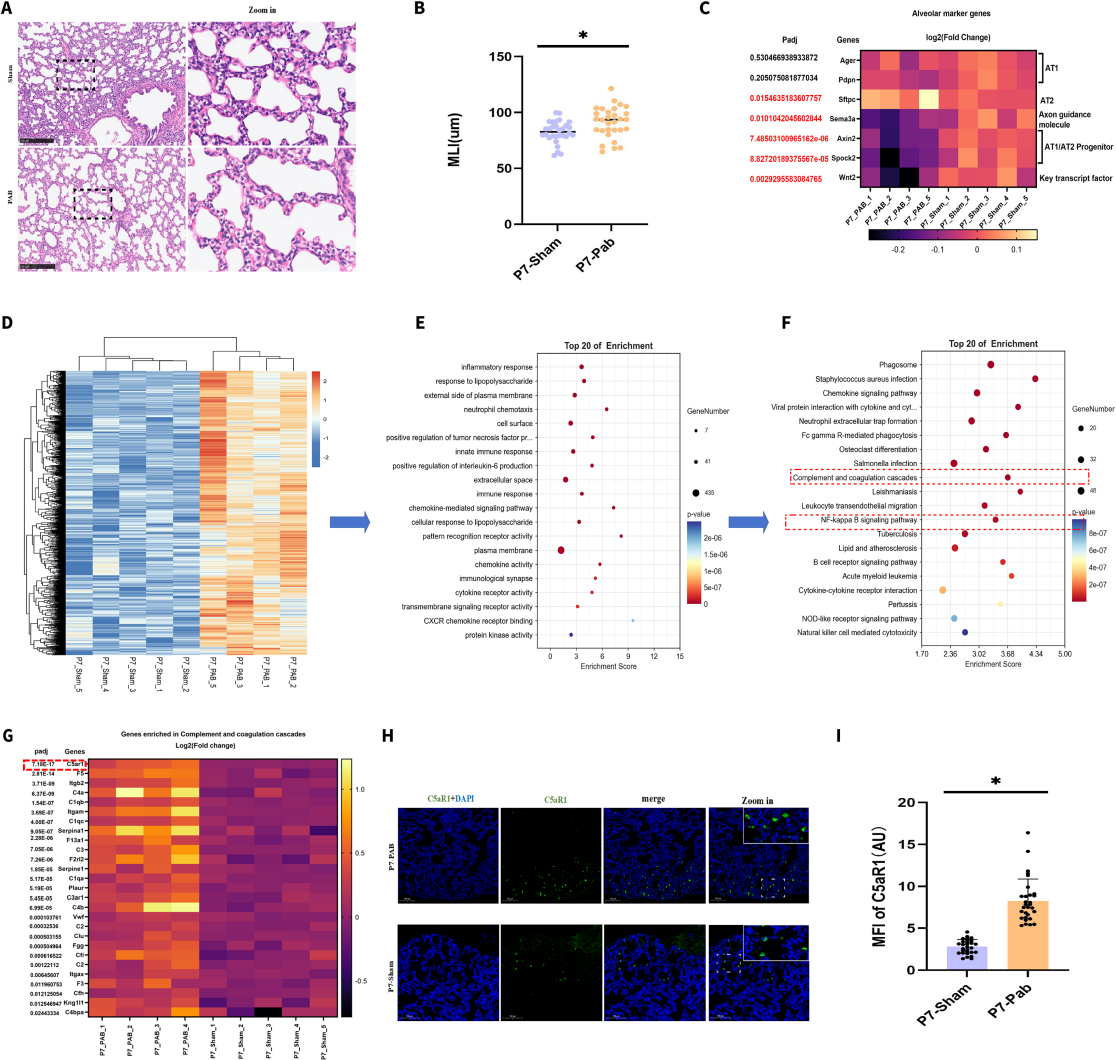
PHypo activates C5aR1 signaling at ALV2. **(A)** Representative hematoxylin and eosin (H&E) staining of lung tissues from P7 PAB and sham rat. **(B)** quantification of mean linear intercept (MLI). **(C)** Bulk RNA-seq of P7 lung tissues. **(D)** Cluster analysis of DEGs at P7. **(E)** GO analysis of upregulated DEGs revealed that the expressions of inflammatory response and immune response were elevated. **(F)** KEGG pathway enrichment analysis specifically identifying complement and downstream NF-κB signaling. **(G)** The enrichment analysis of the complement pathways indicated C5aR1 exhibited the most significant differential expression in the PAB group. **(H)** Representative C5aR1 staining in the sham and PAB groups. C5aR1 (Green); DAPI (Blue). **(I)** Quantification of C5aR1 intensity. *: P ≤ 0.05.

The ALV2 stage represents a critical window of active alveolar septation and heightened developmental plasticity, during which, any hemodynamic abnormality or perturbation of the microenvironment may readily lead to irreversible structural defects manifesting as alveolar simplification. To capture the early molecular events of PHypo-induced injury, we first focused our analysis on P7 (ALV2). (**Figures 1A, B**, indicated with ①). The results showed that PHypo had already induced significant alveolar hypoplasia by P7 (**Figures 2A, B**). RNA-seq revealed upregulation of type II alveolar cell (AT2) markers, alongside downregulation of type I alveolar cell (AT1)/AT2 progenitor markers, the axon guidance molecule SEMA3a, and key alveologenesis-related transcription factors in the PAB group when compared to the sham group (**Figure 2C**; **Supplementary Figures S1A, B**). Downregulated DEGs (PAB vs Sham) enrichment analysis demonstrated that PHypo induced pronounced enrichment of axon guidance and its associated terms (cell migration and focal adhesion) (**Supplementary Figures 1C–E**).

In contrast, enrichment analysis of upregulated DEGs (PAB vs Sham) showed that PHypo induced marked immune activation, with KEGG pathway enrichment analysis specifically identifying complement and downstream NF-κB signaling (**Figures 2D–F**). Among complement pathway genes, C5aR1 exhibited the most significant differential expression in the PAB group when compared to the sham group (padj = 7.18×10^-^¹_7_; **Figure 2G**), and immunofluorescence confirmed elevated C5aR1 protein in the PAB group (**Figures 2H, I**).

**Figure 3:**
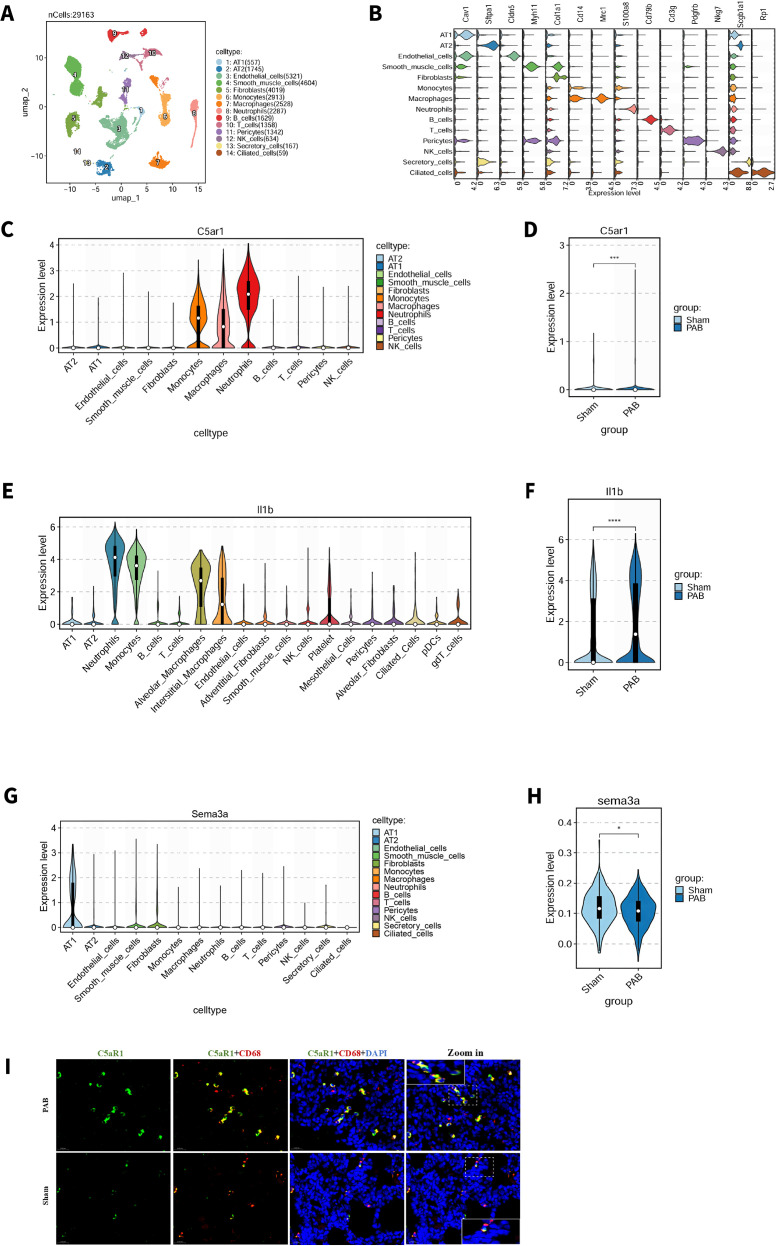
Single-cell RNA sequencing of P7 lungs. **(A)** Cell cluster annotation. **(B)** Violin plot displaying. **(C)** C5aR1 expression distribution. **(D)** Expression of C5aR1 in PAB and Sham. **(E)** IL-1β expression distribution. **(F)** Expression of IL-1β in PAB and Sham. **(G)** Sema3a expression distribution. **(H)** Expression of Sema3a in PAB and Sham. **(I)** Subcellular localization of C5aR1 in macrophages. *: P≤0.05; ***: P≤0.001; ****: P≤0.0001.

To further investigate which cell types express C5aR1 and produce IL-1β, we performed single-cell RNA sequencing analysis (**Figures 3A, B**). The results revealed that monocytes and macrophages were among the top three cell types with the highest expression levels of C5aR1 and IL-1β, and showed higher expression in the PAB group than in the sham group (**Figures 3C–F**). These findings are largely consistent with current understanding (16). Interestingly, however, neutrophils represented the cell subset with the highest C5aR1 and IL-1β expression, suggesting that neutrophils may also play an important role—a finding that challenges conventional views and warrants further detailed investigation.

In contrast, SEMA3a was predominantly expressed in AT1 cells, with significantly lower expression in the PAB group than in the sham group (**Figures 3G, H**). In macrophages, C5aR1 was primarily localized in the cytoplasm (**Figure 3I**).

Collectively, these findings indicate that at the ALV2 stage, C5a likely disrupts alveolar development at the ALV2 stage by suppressing axon guidance mechanisms in the PAB group.

### 3.2 PHypo-induced alveolar hypoplasia is further aggravated at ALV4 (P14)

**Figure 4:**
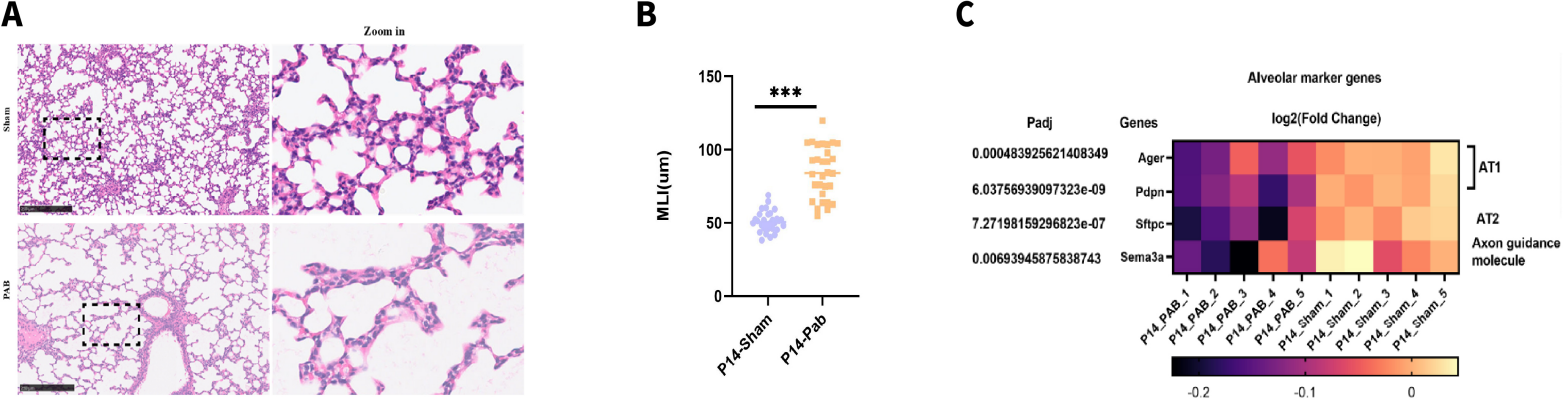
PHypo-induced alveolar hypoplasia at ALV4. **(A)** Representative hematoxylin and eosin (H&E) staining of lung tissues from P14 PAB and sham piglets. **(B)** Quantification of mean linear intercept (MLI). **(C)** Bulk RNA-seq of P14 lung tissues. ***: P ≤ 0.001.

To determine the long-term consequences of early C5aR activation, we assessed alveolar structure at P14 (ALV4) (**Figures 1A, B**, marked with ②). Histological assessment at the ALV4 (P14) demonstrated marked alveolar hypoplasia in the PAB group (**Figures 4A, B**). Bulk RNA-seq of P14 lung tissues showed significant downregulation of AT1 markers (Ager, Pdpn), AT2 marker (Sftpc), and Sema3a in the PAB group (**Figure 4C**). Gene set enrichment analysis of downregulated differentially expressed genes (DEGs) (PAB vs Sham) revealed that there were enriched terms associated with key alveolar developmental processes, including axon guidance, cell migration, focal adhesion (**Supplementary Figures S2A–E**). These findings were corroborated by immunofluorescence showing reduced expression of AT1, AT2, and axon guidance markers in the PAB group (**Supplementary Figures S3A–F**).

Upregulated DEGs (PAB vs Sham) enrichment analysis indicated there were immune response activation in the PAB group (**Supplementary Figures S2F–H**), consistent with prior reports (1, 2).

### 3.3 C5a antagonism rescues PHypo-induced alveolar hypoplasia

**Figure 5:**
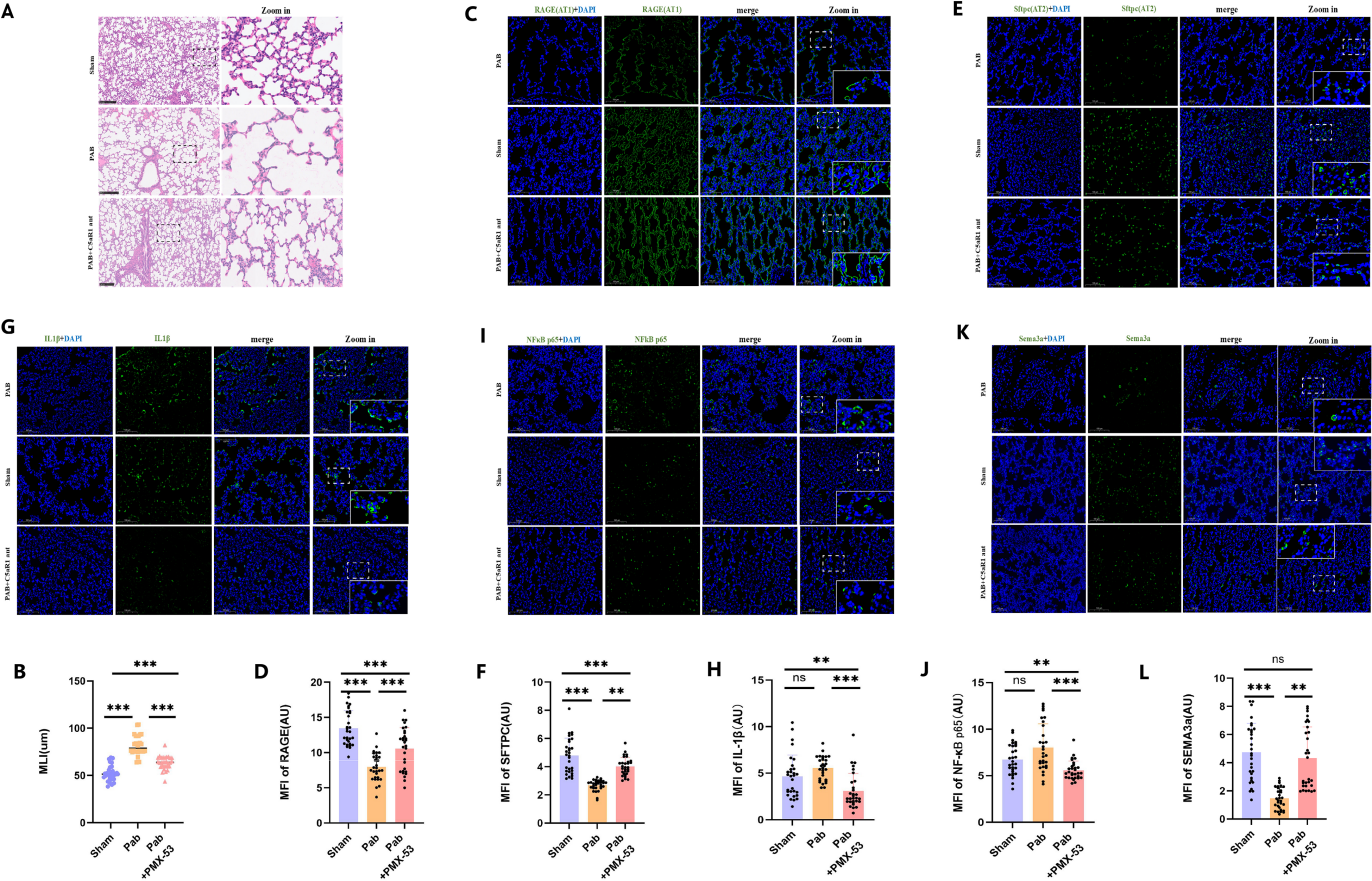
C5aR1 antagonism rescues PHypo-induced alveolar hypoplasia. **(A)** Representative hematoxylin and eosin (H&E) staining of lung tissues from P14 PAB and sham rat. **(B)** Quantification of mean linear intercept (MLI). **(C)** Representative Rage (AT1) staining in the sham and PAB groups. Rage (Green); DAPI (Blue). **(D)** Quantification of Rage intensity. **(E)** Representative Sftpc (AT2) staining in the sham and PAB groups. Sftpc (Green); DAPI (Blue). **(F)** Quantification of Sftpc intensity. **(G)** Representative IL1β staining in the sham and PAB groups. IL-1β (Green); DAPI (Blue). **(H)** Quantification of IL-1β intensity. **(I)** Representative NF-κB p65 staining in the sham and PAB groups. NF-κB p65 (Green); DAPI (Blue). **(J)** Quantification of NF-κB p65 intensity. **(K)** Representative Sema3a staining in the sham and PAB groups. Sema3a (Green); DAPI (Blue). **(L)** Quantification of Sema3a intensity. **: P ≤ 0.01; ***: P ≤ 0.001; ns: P > 0.05.

To establish C5a’s role in PHypo-induced alveolar hypoplasia and explore its therapeutic relevance, neonatal PHypo rats received continuous C5aR1 antagonist (C5aR1 ant) PMX53 treatment for 14 days (**Figures 1A, B**, marked with ③). The results demonstrated that C5aR1 ant significantly increased alveolar number (**Figures 5A, B**). Furthermore, C5aR1 ant treatment enhanced the immunofluorescence signal intensity of both AT1 and AT2 cells in PHypo lungs (**Figures 5C–F**). These findings not only confirm C5a’s critical role in PHypo-induced alveolar hypoplasia but also suggest the potential clinical applicability of C5aR1 antagonists.

Immunofluorescence analysis of C5a downstream molecules IL-1β and NF-κB demonstrated that C5aR1 antagonist treatment significantly reduced IL-1β expression (**Figures 5G, H**) and NF-κB activation (**Figures 5I, J**). Consequently, expression of SEMA3a was markedly upregulated (**Figures 5K, L**). Together, these results establish the C5a→IL-1β→NF-κB→SEMA3a axis as the fundamental mechanism mediating PHypo-induced alveolar hypoplasia.

### 3.4 IL-1β inhibition rescues PHypo-induced alveolar hypoplasia

**Figure 6:**
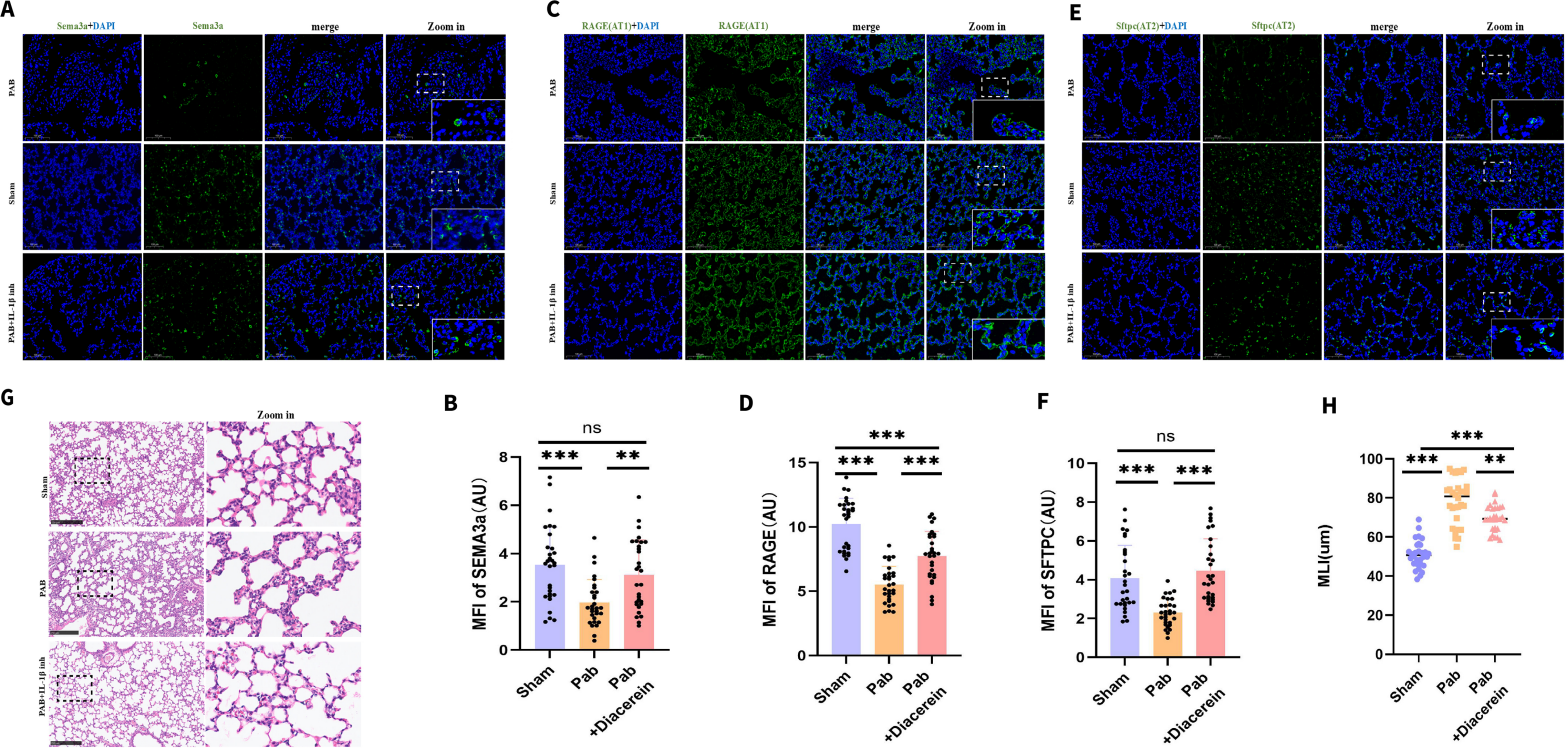
IL-1β inhibition rescues PHypo-induced alveolar hypoplasia. **(A)** Representative Sema3a staining in the sham and PAB groups. Sema3a (Green); DAPI (Blue). **(B)** Quantification of SEMA3a intensity. **(C)** Representative Rage (AT1) staining in the sham and PAB groups. Rage (Green); DAPI (Blue). **(D)** Quantification of Rage intensity. **(E)** Representative Sftpc (AT2) staining in the sham and PAB groups. Sftpc (Green); DAPI (Blue). **(F)** Quantification of Sftpc intensity. **(G)** Representative hematoxylin and eosin (H&E) staining of lung tissues from P14 PAB and sham rat. **(H)** Quantification of mean linear intercept (MLI). **: P ≤ 0.01; ***: P ≤ 0.001; ns: P > 0.05.

To further elucidate the role of C5a in PHypo-induced alveolar hypoplasia, we treated neonatal PHypo rats with an IL-1β inhibitor, which significantly suppressed IL-1β expression (**Supplementary Figures S4A, B**) and NF-κB activation (**Supplementary Figures S4C, D**), leading to subsequent upregulation of the axon guidance molecule SEMA3a (**Figures 6A, B**) that ultimately restored AT1/AT2 cell populations (**Figures 6C–F**) and improved alveolar numbers (**Figures 6G, H**), thereby demonstrating the therapeutic potential of IL-1β inhibition and further validating the crucial role of the C5a-IL-1β pathway in PHypo-induced alveolar hypoplasia.

### 3.5 Clinical correlation analysis

**Figure 7:**
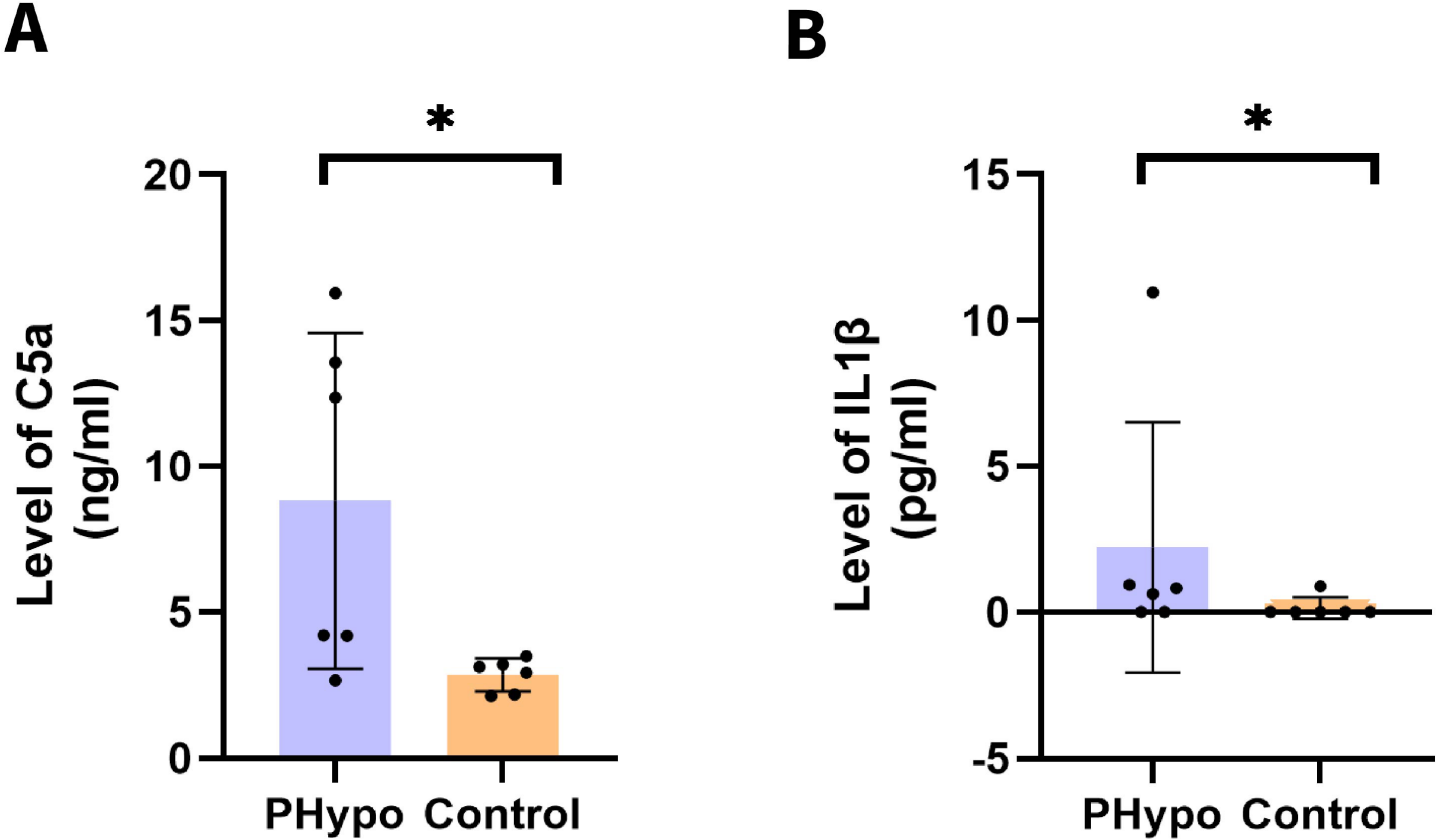
Upregulated C5a and IL-1β concentration in pediatric PHypo patients. **(A)** Comparison of serum C5a levels between children with and without pulmonary hypoperfusion. **(B)** Comparison of serum IL-1β levels between children with and without pulmonary hypoperfusion. *: P ≤ 0.05.

To enhance the translational relevance of our findings, we analyzed serum samples from pediatric PHypo patients. ELISA quantification revealed significantly elevated circulating levels of both C5a (4-fold increase, p=0.03) and IL-1β (2-fold increase, p=0.022) compared to age-matched controls (**Figures 7A, B**). These clinical observations strongly support the therapeutic potential of targeting the C5a/IL-1β axis for ameliorating PHypo-induced alveolar hypoplasia in human patients.

## 4 Discussion

This study establishes the C5a-IL-1β-NF-κB-SEMA3a signaling axis as a central mechanism driving alveolar hypoplasia in pulmonary hypoperfusion (PHypo), with key translational implications for CHD management. Our principal findings demonstrate that: (1) PHypo triggers early complement cascade activation (peaking at ALV2/P7), with C5aR1 as the most significantly upregulated gene; (2) C5a-mediated IL-1β induction suppresses SEMA3a via NF-κB phosphorylation, disrupting axon guidance-dependent alveologenesis; (3) Pharmacological blockade of C5aR1 (PMX53) or IL-1β rescues alveolarization in neonatal rats; and (4) Pediatric PHypo patients exhibit profoundly elevated serum C5a and IL-1β (4-fold and 2-fold, respectively), clinically validating this mechanism.

The work provides three significant advances: First, it resolves the paradox of how immune activation impairs alveolar development by linking sterile inflammation (C5a) to axon guidance pathways (SEMA3a). Second, it identifies the developmentally vulnerable ALV2 window (P4-P7) as the critical period for therapeutic intervention. Third, it demonstrates dual-target therapeutic efficacy using clinically relevant inhibitors (C5aR1/IL-1β), with rescue effects.

These findings directly address the unmet clinical need of progressive exercise intolerance in CHD survivors. By pinpointing C5a/IL-1β as druggable targets upstream of alveolar simplification, we provide a mechanistic rationale for: Repurposing existing immunomodulators (e.g., IL-1β antagonists like anakinra) for neonatal PHypo, Precision timing of interventions during the ALV2 developmental window, Biomarker-guided therapy using serum C5a/IL-1β levels to identify high-risk patients.

### 4.1 Limitations

While the PAB model replicates human RVOTO hemodynamics, three constraints warrant consideration: (1) Species differences in alveolar maturation: Rat ALV2-ALV4 (P4-P18) spans ~3 weeks versus human alveolarization (0–7 years), limiting direct temporal extrapolation. (2) Although single-cell sequencing results initially suggest that C5aR1 and IL-1β originate from monocytes, macrophages, and neutrophils, it remains unclear which cell type plays a dominant role or whether coordinated interactions exist among these populations. Further investigation using cell-specific C5aR1 knockout is warranted to establish definitive causal relationships. (3) Therapeutic scope: PMX53’s peptide structure limits bioavailability; non-peptide C5aR1 antagonists should be evaluated.

Four strategic paths emerge: Larger animal studies using fetal lamb/piglet CHD models to validate therapeutic windows in physiologically relevant timeframes. Cell-specific knockout models (e.g., C5ar1δ[mac] vs. δ[AT1]) to dissect immune-epithelial crosstalk. Clinical translation: Phase I trials of IL-1β inhibition in RVOTO patients using alveolarization metrics (CT densitometry) as endpoints. Mechanistic expansion: Investigate C5a’s role in other hypoperfusion contexts (e.g., bronchopulmonary dysplasia).

## 5 Conclusions

Our work illuminates PHypo-induced alveolar hypoplasia as a sterile inflammation-driven disorder amenable to immunomodulation. By defining the C5a→IL-1β→NF-κB→SEMA3a axis and demonstrating rescue via targeted inhibition, we provide both mechanistic insight and actionable therapeutic strategies. These advances hold significant promise for improving long-term pulmonary outcomes in the growing population of CHD survivors.

## Data Availability

The data generated in this study are available from the corresponding author upon reasonable request. The bulk RNA-seq data have been deposited in the GEO database (https://www.ncbi.nlm.nih.gov/geo) under accession No. GSE201522 and No. GSE206972, respectively.

## Supplementary material

Supplementary Figure 1 RNA-seq analysis of P7 lungs. **(A)**. Volcano map of differentially expressed genes (DEGs) of postnatal alveolar development in the PAB and Sham condition (P7_PAB vs.P7_Sham). **(B-E)**. Downregulated DEGs (PAB vs Sham) enrichment analysis demonstrated that PHypo induced more pronounced enrichment of axon guidance and its associated terms (cell migration and focal adhesion) at P7.

Supplementary Figure 2 RNA-seq analysis of P14 lungs. **(A)**. Volcano map of differentially expressed genes (DEGs) of postnatal alveolar development in the PAB and Sham condition. (P14_PAB vs. P14_Sham). **(B-H)**. Gene set enrichment analysis of downregulated differentially expressed genes (DEGs) (PAB vs Sham) revealed that there were enriched terms associated with key alveolar developmental processes, including axon guidance, cell migration, focal adhesion.

Supplementary Figure 3 Immunofluorescence verification at P14 (ALV4) **(A)**. Representative Rage (AT1) staining in the sham and PAB groups. Rage (Green); DAPI (Blue). **(B)**. Representative Sftpc (AT2) staining in the sham and PAB groups. Sftpc (Green); DAPI (Blue). **(C)**. Quantification of Rage intensity. **(D)**. Quantification of Sftpc intensity. **(E)**. Representative Sema3a staining in the sham and PAB groups. SEMA3a (Green); DAPI (Blue). **(F)**. Quantification of SEMA3 intensity.

Supplementary Figure 4 Expression of SEMA3a/IL-1β after IL-1β inhibition. **(A)**. Representative IL-1β staining in the sham and PAB groups. IL-1β (Green); DAPI (Blue). **(B)**. Quantification of IL-1β intensity. **(C)**. Representative NF-κB staining in the sham and PAB groups. NF-κB p65 (Red); DAPI (Blue). S2D. Quantification of NF-κB p65 intensity.
