# Supplementary figures and images for: Complement C5a receptor 1 antagonist attenuates alveolar hypoplasia induced by pulmonary hypoperfusion and its underlying mechanisms
Source: Front Immunol. 2026 May 11;17:1802250. doi: 10.3389/fimmu.2026.1802250 (PMC13199021; doi:10.3389/fimmu.2026.1802250)

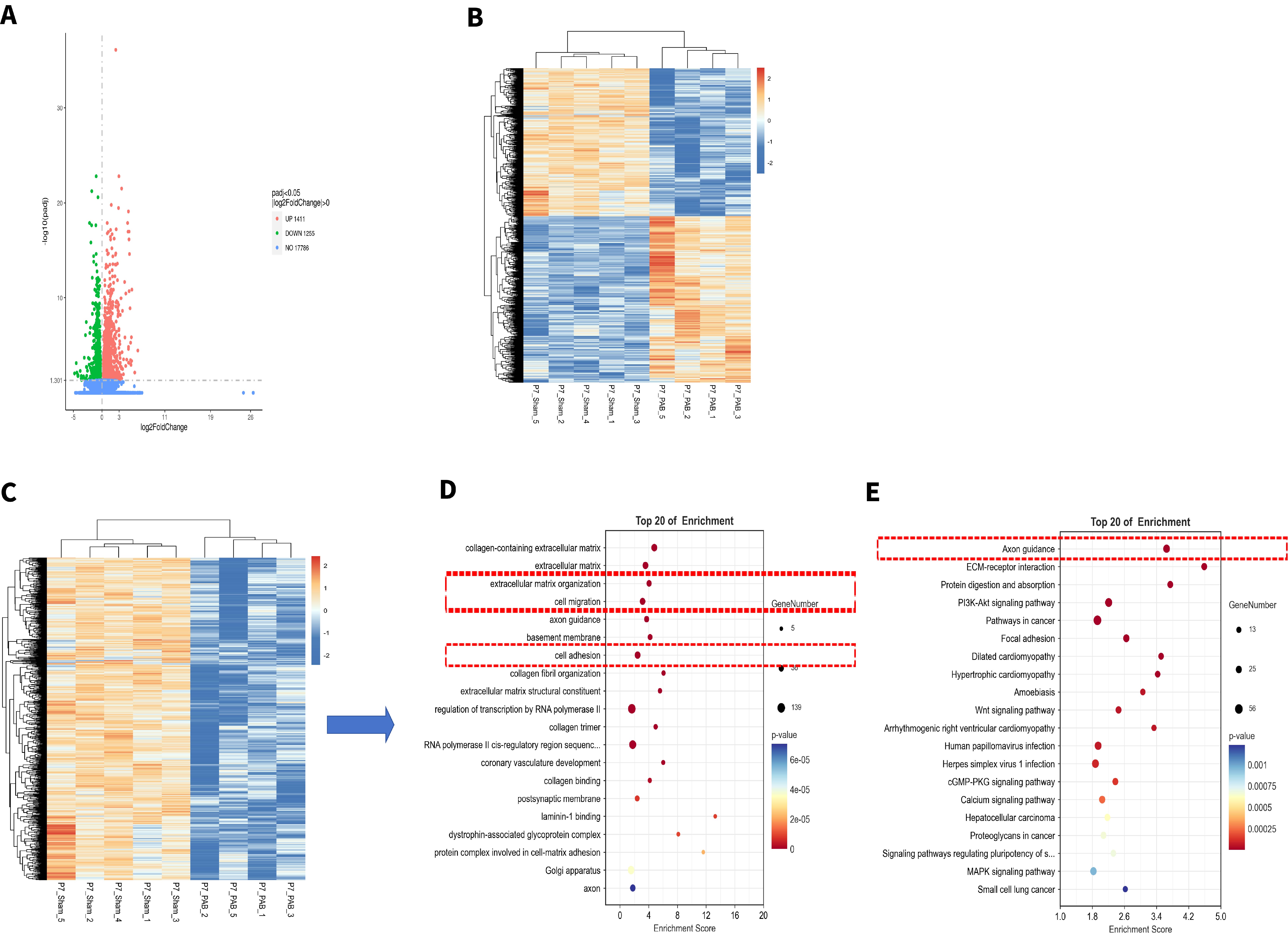

Supplement: Supplementary Figure 1 — RNA-seq analysis of P7 lungs. (A). Volcano map of differentially expressed genes (DEGs) of postnatal alveolar development in the PAB and Sham condition (P7_PAB vs.P7_Sham). (B-E). Downregulated DEGs (PAB vs Sham) enrichment analysis demonstrated that PHypo induced more pronounced enrichment of axon guidance and its associated terms (cell migration and focal adhesion) at P7. [file Image1.jpeg]

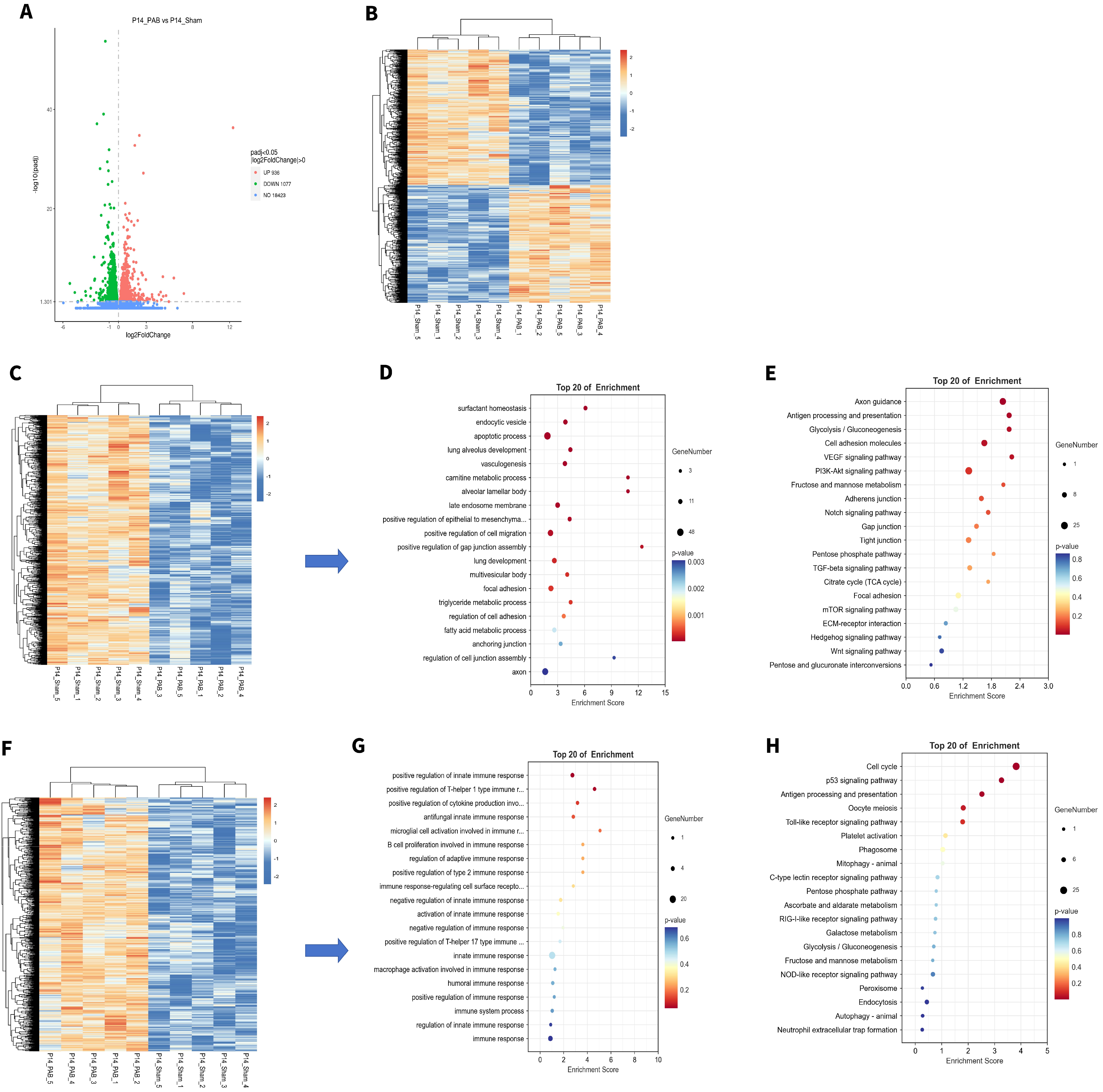

Supplement: Supplementary Figure 2 — RNA-seq analysis of P14 lungs. (A). Volcano map of differentially expressed genes (DEGs) of postnatal alveolar development in the PAB and Sham condition. (P14_PAB vs. P14_Sham). (B-H). Gene set enrichment analysis of downregulated differentially expressed genes (DEGs) (PAB vs Sham) revealed that there were enriched terms associated with key alveolar developmental processes, including axon guidance, cell migration, focal adhesion. [file Image2.jpeg]

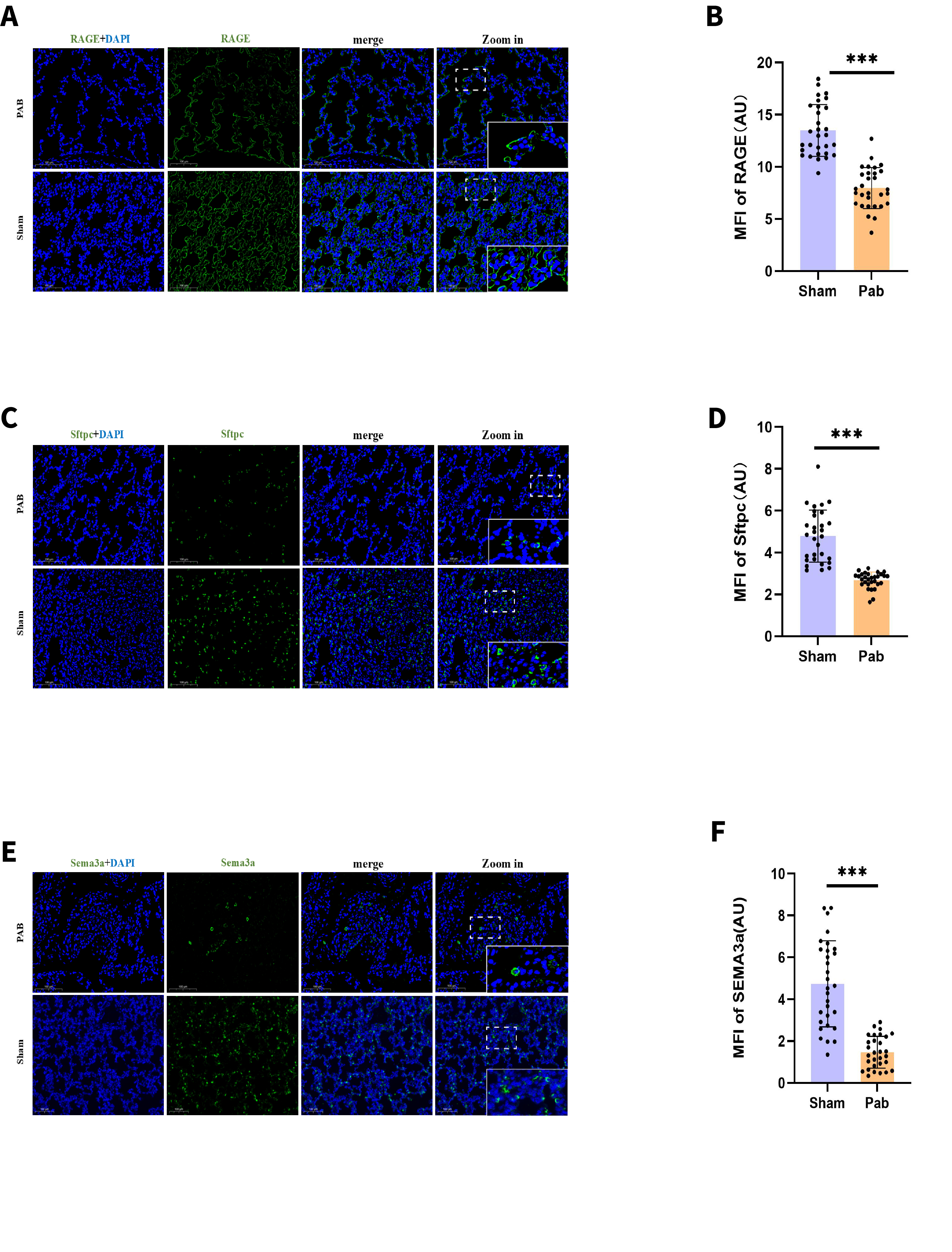

Supplement: Supplementary Figure 3 — Immunofluorescence verification at P14 (ALV4) (A). Representative Rage (AT1) staining in the sham and PAB groups. Rage (Green); DAPI (Blue). (B). Representative Sftpc (AT2) staining in the sham and PAB groups. Sftpc (Green); DAPI (Blue). (C). Quantification of Rage intensity. (D). Quantification of Sftpc intensity. (E). Representative Sema3a staining in the sham and PAB groups. SEMA3a (Green); DAPI (Blue). (F). Quantification of SEMA3 intensity. [file Image3.jpeg]

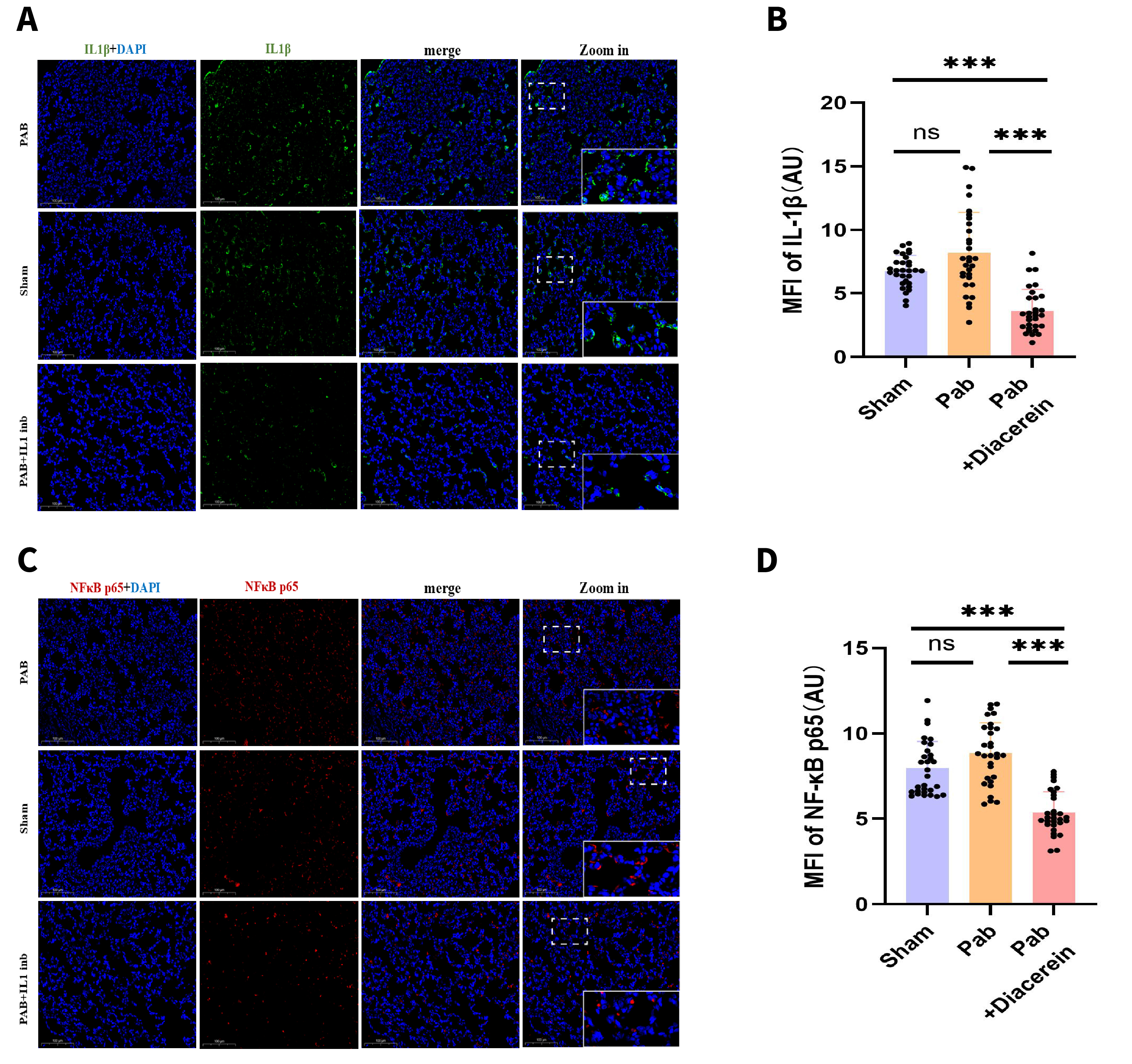

Supplement: Supplementary Figure 4 — Expression of SEMA3a/IL-1β after IL-1β inhibition. (A). Representative IL-1β staining in the sham and PAB groups. IL-1β (Green); DAPI (Blue). (B). Quantification of IL-1β intensity. (C). Representative NF-κB staining in the sham and PAB groups. NF-κB p65 (Red); DAPI (Blue). S2D. Quantification of NF-κB p65 intensity. [file Image4.jpeg]
