# Supplementary material for: Complement C5a receptor 1 antagonist attenuates alveolar hypoplasia induced by pulmonary hypoperfusion and its underlying mechanisms
Source: Front Immunol. 2026 May 11;17:1802250. doi: 10.3389/fimmu.2026.1802250 (PMC13199021; doi:10.3389/fimmu.2026.1802250)
Supplement: Supplementary file 5 [file Table1.docx]

| **Abtibodies** | **Manufacturer** | **Product No.** |
| --- | --- | --- |
| PMX53 | MedchemExpress | 219639-75-7 |
| Diacerein | APExBiO | 13739-02-1 |
| Anti C5aR1 | Proteintech | 21316-1-AP |
| Anti IL-1β | Proteintech | 26048-1-AP |
| Anti NF-κB p65 | Proteintech | 82335-1-RR |
| Anti Sema3a | Santa Cruz | sc-74555 |
| Anti RAGE | abcam | ab21171 |
| Anti SFTPC  Anti CD68 | Thermo Fisher  abcam | PA5-71680  ab283654 |

**Supplemental Table S1 Antibodies and Reagents**
